# Recent XAS studies into Homogeneous metal catalyst in fine chemical and pharmaceutical syntheses

**DOI:** 10.1186/s13065-015-0103-6

**Published:** 2015-06-18

**Authors:** Grant J Sherborne, Bao N Nguyen

**Affiliations:** Institute of Process Research & Development, School of Chemistry, University of Leeds, Woodhouse Lane, Leeds, LS2 9JT UK

**Keywords:** X-ray absorption spectroscopy, Homogeneous catalysis, Mechanism

## Abstract

A brief review of studies using X-ray Absorption Spectroscopy (XAS) to investigate homogeneous catalytic reactions in fine chemical and pharmaceutical context since 2010 is presented. The advantages of the techniques over traditional lab-based analytical tools, particularly when NMR spectroscopy fails to deliver mechanistic insights, are summarised using these examples. A discussion on the current limitations of the techniques and challenges in the near future is also included.

**Graphical abstract:**
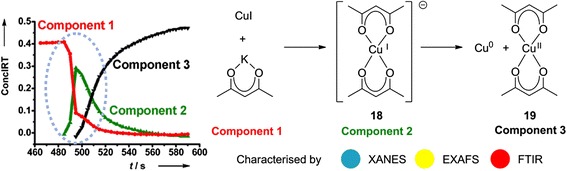
A minireview of recent developments in application of X-ray Absorption Spectroscopy as an effective mechanistic tool to synthetic catalytic reactions relevant to fine chemical and pharmaceutical syntheses.

A minireview of recent developments in application of X-ray Absorption Spectroscopy as an effective mechanistic tool to synthetic catalytic reactions relevant to fine chemical and pharmaceutical syntheses.

XAS has been very successfully applied as a technique to study heterogeneous catalysts [1, 2]. Electronic and structural information can be extracted from the absorption edge region (X-ray Absorption Near Edge Spectroscopy or XANES) and the scattering pattern (Extended X-ray Absorption Fine Structure Spectroscopy or EXAFS), respectively (Fig. 1) [3]. The major advantage of the technique, in a catalysis context, is that the metal centre of the catalyst can be selectively observed under turnover conditions while ignoring organic species in the system.

**Fig. 1:**
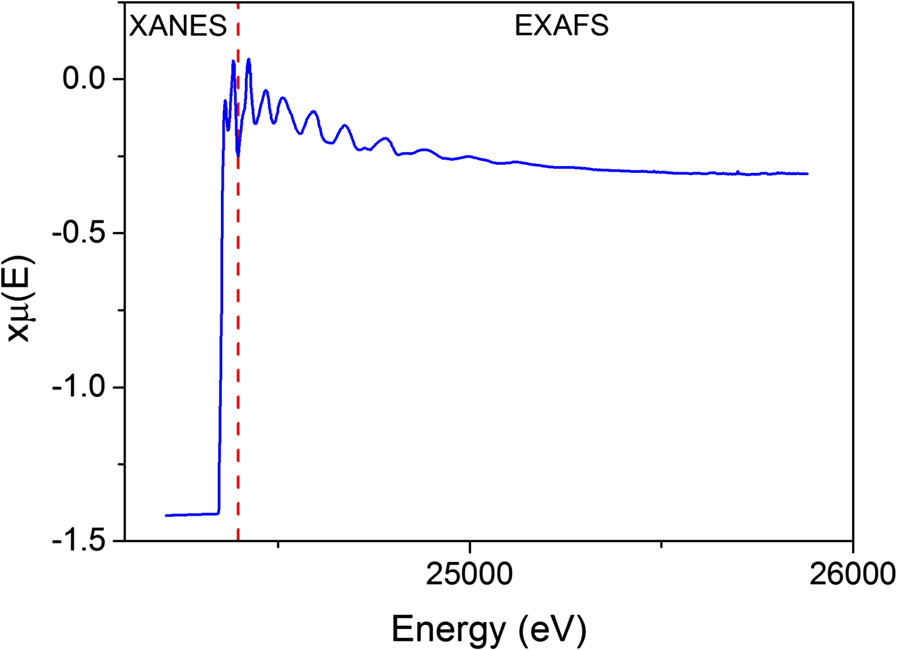
Example of XAS output which includes **a**) XANES region which contains information on the oxidation state and electronic structure of the observed atom and **b**) EXAFS region which contains information on the bonding environment around the atom

Applications of XAS to homogeneous catalysis in liquid phase, however, are limited. This is due firstly to the inherently poor signal-to-noise ratio, a result of low concentration of catalyst and background absorption by organic materials. Another, less obvious, obstacle is the required effort in developing a suitable sample environment for each study, which can improve signal quality. This requires good understanding of the physics of the technique, and an additional level of commitment to planning and testing of sample environment for synthetic researchers. Studies at low energy (<9 keV, e.g. Cu, Fe, Ni, Co) are particularly difficult in solution as the solvent can significantly absorb radiation. Consequently, sample environments vary from stopped-flow cuvettes [4] to PTFE [5, 6] and PEEK cells [7] with Kapton® windows. In addition, time resolved in situ studies sometimes suffer from sample decomposition by radiation, which necessitates spectroscopic flow-cells through which reaction mixtures can be pumped continuously. These flow-cells also enable steady state measurements and time resolution through changes to residence time and flow-rate [8, 9]. When such problems are overcome, XAS has been demonstrated to be a highly effective method, and in some cases the only appropriate method, to answer many mechanistic questions in homogeneous catalysis.

In this mini-review, we highlighted recent successful XAS studies of homogeneous catalysts since 2010. As we aim to encourage interest from the synthetic community, only discrete molecular catalysts and nanopaticles, which catalyse organic transformations in fine chemical/pharmaceutical context, will be included. Studies using static samples will be discussed first, before the more technologically challenging in situ studies under turnover conditions. Readers who seek more detailed information on the techniques and its practical aspects should refer to comprehensive reviews of the fields by Eisenberger [10], Evans [11], and Lamberti [3].

A simple and effective study into the role of chiral phosphate counterions in gold-catalysed reaction was reported by Nguyen et al. [12]. In their study, Au L-edge EXAFS spectrum of [Ph_3_PAu][OP(=O)-BINOL] (1) in toluene clearly indicated a short Au-OP(=O)-BINOL bond (2.02 Å) at resting state in solution (Fig. 2). This suggests that the chiral phosphate counterion can act as a chiral ligand in many enantioselective gold-catalysed reactions [13–15].

**Fig. 2:**
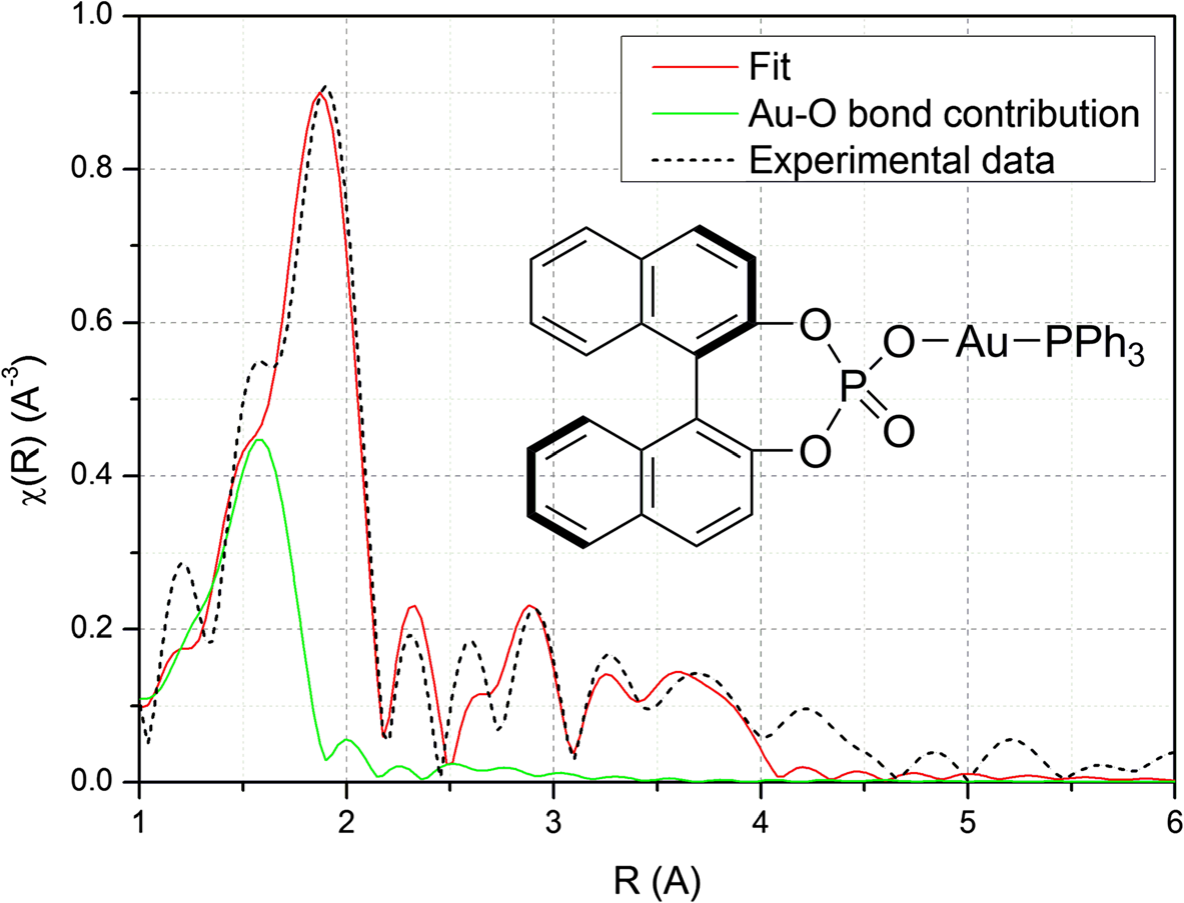
EXAFS spectrum and fitting for [Ph_3_PAu][OP(=O)-BINOL] (1) (reproduced with permission from ACS Publications)

The oxidation state of gold catalyst in an Au-catalysed cyclobenzannulation reaction, a common debate in homogenous gold catalysis [16], was also studied by Nguyen (Scheme 1) [12]. Monitoring stoichiometric steps of the catalytic cycle using XANES indicated that the majority of Au-species are Au(III), based on the absorption edge position which is oxidation state sensitive, and a stoichiometry different from 1 : 1 between AuCl_3_ and the substrate. This led to the detection and characterisation by ^1^H NMR and HRMS of novel intermediates four and five, which reacts with phenylacetylene to give the final product.

**Scheme 1:**
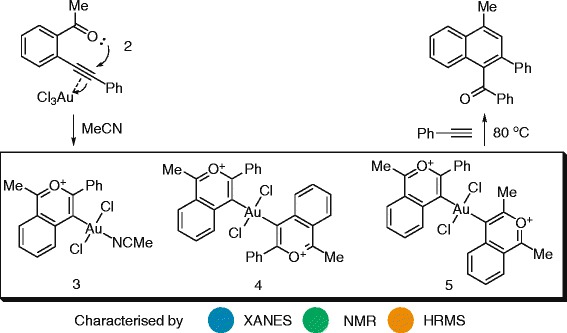
Novel intermediates detected by XAS in a Au-catalysed cyclobenzannulation

Hashmi and Bertagnolli also employed XAS to investigate the oxidation state of the gold catalyst in an Au-catalysed oxidative esterification [6]. No Au-Au bond was detected by Au L-edge EXAFS, supporting the homogeneous nature of the catalyst. The oxidation state of gold species in these samples was determined through *Linear Combination Analysis* (LCA) of XANES spectra against those of HAuCl_4_/MeCN and AuCl as standards. The results indicated a Au(III) : Au(I) ratio of 88 : 12 at the beginning of the reaction, which changed to 14 : 86 after 24 h when the oxidant is consumed (Scheme 2).

**Scheme 2:**
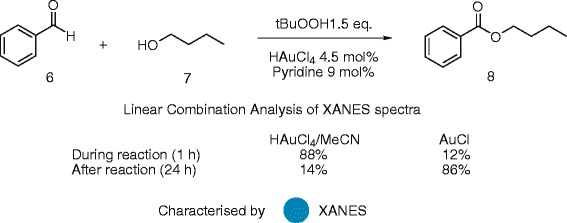
Au-catalysed oxidative esterification and catalyst oxidation state during and after reaction

Fe-catalysed cross-coupling reactions are a rapidly growing field of research in which mechanistic insights are difficult to obtain due to the paramagnetic nature of many Fe-species which precludes NMR techniques. Bauer and co-workers reported the use of XAS to study the mechanism of reaction between aryl halides and Grignard reagents (Scheme 3) [17]. Titration of the pre-catalyst Fe(acac)_3_ with PhMgCl (10) was monitored using Fe K-edge XANES spectra, which led to assignment of a Fe(I) active species. However, EXAFS data suggested the formation of nanoparticles (13 ± 2 atoms), i.e. Fe-Fe bonds, on which the Fe(I) centres on the surface can undergo oxidative addition with aryl halides to become Fe(III) centres. The average observed oxidation state of Fe was determined to be +1.7 under reaction conditions based on the position of the absorption edge.

**Scheme 3:**
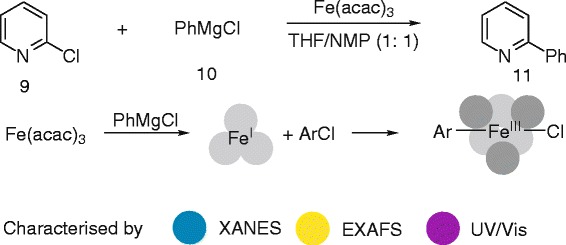
Fe-catalysed coupling reaction between aryl halides and Grignard reagents and its mechanism

The above study also demonstrated that the boundary between homogeneous and heterogeneous catalysis can sometime be blurred in transition metal catalysed reactions [18, 19]. Another investigation by Fairlamb and Lee on a Suzuki-Miyaura reaction catalysed by Pd-nanoparticles differentiated between catalysis on the surface of Pd-nanoparticles and catalysis by leached Pd atoms/colloids in solution [20]. Ex situ and in situ measurements at the Pd-K edge were used to link catalytic activity to defect sites on the 1.8 nm Pd nanoparticles, comprised of 236 Pd atoms, which was supported by kinetics, TEM and Hg poisoning experiments. This is the first incontrovertible evidence that this type of reaction can operate under heterogeneous conditions.

The Chan-Lam reaction, a popular C-N coupling reaction under very mild conditions, was investigated by Tromp et al. using time-resolved in situ XAS and UV/Vis spectroscopy [4]. The use of energy dispersive, rather than energy scanning, XAS allowed collection of XANES and EXAFS spectra in seconds/min timescale. Stable intermediates of the catalytic cycle were characterised, using a combination of XANES, EXAFS and UV/Vis spectroscopy, in step-wise fashion using [Cu(μ_2_-OH)(TMEDA)]_2_Cl_2_ (12) as catalyst (Scheme 4). Simultaneous injection of all reaction components in stoichiometric amounts led to formation of a monomeric Cu(I) active catalytic species. As the product *N*-phenylimidazole (13) is formed, the Cu(II) pre-catalyst was restored. The proposed short-lived Cu(III) intermediate 15 of this reaction, however, could not be observed.

**Scheme 4:**
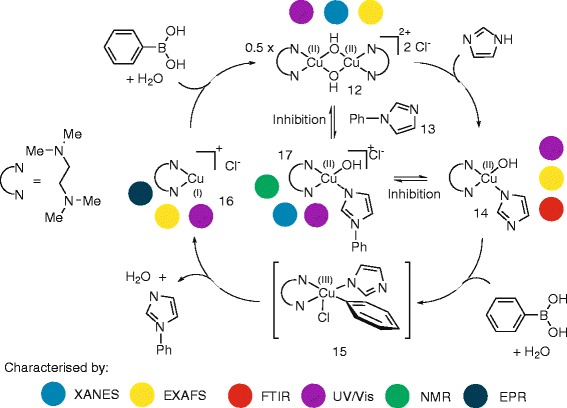
Catalytic cycle of Cu(II)-catalysed arylation of imidazole and phenylboronic acid

Lei et al. studied a less well-known but synthetically useful Cu-catalysed C-C coupling reaction between acetylacetone and an aryl halide, using a combination of in situ FTIR and Cu K-edge XAS [7]. Acetylacetone was found to act both as a ligand for the Cu-catalyst and a coupling partner. Importantly, in situ Cu K-edge XANES and EXAFS studies of a stoichiometric reaction between K(acac) and CuI (Scheme 5) led to identification of product [Cu(I)(acac)_2_]^−^ 18, which disproportionates to [Cu(II)(acac)_2_] 19, confirmed by FT-IR, and Cu(0) within 10 min in a catalyst deactivation pathway.

**Scheme 5:**
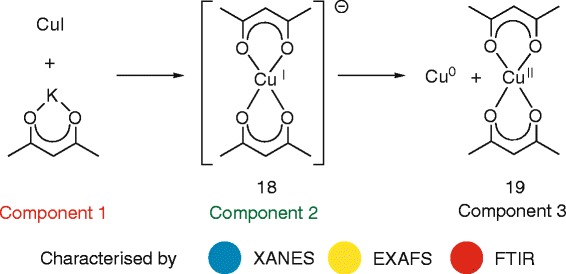
Kinetic profile of the stoichiometric reaction between K(acac) and CuI, and the corresponding catalyst deactivation pathway. (partially reproduced with permission from ACS Publications)

Tanaka and Shishido recently reported an in situ time-resolved Ni K-edge XAS study of a reaction between [Ni(bipy)(COD)] and PhBr giving 1,1’-biphenyl as product within an impressive timescale of 100 s [21]. The starting complex [Ni(bipy)(COD)], the oxidative addition product [Ni(bipy)(Ph)(Br)(DMF)_2_], and the by-product [Ni(bipy)Br_2_(DMF)_2_] were assigned and characterised by EXAFS fitting. A Ni-Br bond length of 2.61 Å and a Ni-Ph bond length of 2.08 Å were determined for [Ni(bipy)(Ph)(Br)(DMF)_2_]. The Ni-Br bond length was shortened to 2.47 Å in the by-product [Ni(bipy)Br_2_(DMF)_2_] after formation of 1,1’-biphenyl. The concentrations of these three major species were extracted by LCA of both XANES and EXAFS spectra of the reaction over time, leading to near identical reaction profiles (Fig. 3). Observed coordination of DMF molecules to Ni explains experimental observation that polar solvents are beneficial in the reaction.

**Fig. 3:**
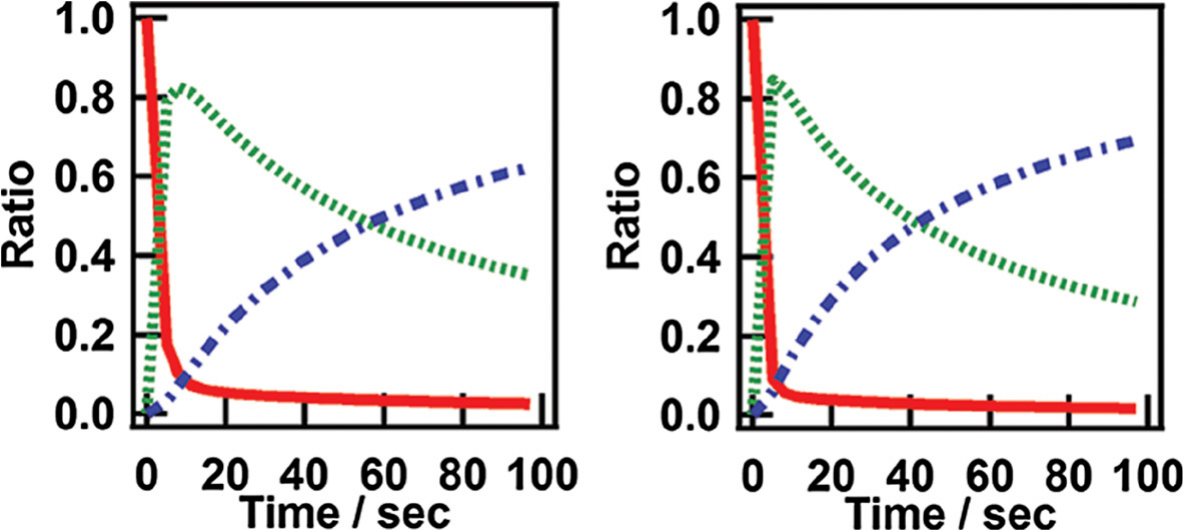
Time course of the concentration of each LCA extracted species (left: XANES; right: EXAFS); red solid line: [Ni(bipy)(COD)]; green dotted line: [Ni(bipy)(Ph)(Br)(DMF)_2_]; blue hashed line: [Ni(bipy)Br_2_(DMF)_2_]. (reproduced with permission from ACS Publications)

Recent advances in XAS include the applications of HERFD-XAS (High-Energy Resolution Fluorescence Detected XAS), [22–24] and RIXS (Resonant Inelastic X-ray Scattering), [25, 26] which give much higher resolution for the edge and pre-edge region. Bauer took advantage of these to improve his prior study of Fe-catalysed Michael addition of a β-diketone to an enone [27]. Higher resolution Fe K-edge XANES data led to a corrected ratio of [FeCl_4_]^−^ : [Fe(L)_2_(H_2_O)_2_]^+^ of 78.6 : 21.4, instead of 50 : 50 as previously determined using standard XANES [28]. A titration experiment at Fe K-edge with Et_3_NBzCl revealed that Cl^−^ poisons the catalyst by converting it to the inactive [FeCl_4_]^−^. This explains the lower catalytic activity observed when FeCl_3_ was used as pre-catalyst instead of Fe(ClO_4_)_3_.

## Conclusions

Applications of XAS to homogeneous catalysis in synthetic context are surprisingly few, given its potential insights on the oxidation state, electronic configuration and coordination environment of the catalyst under turnover conditions. However, a number of studies in recent years have overcome its technical hurdles to demonstrate its power over a wide range of transition metal catalysed reactions. Whilst characterisation of intermediates using XAS alone is difficult, this can often be solved by complementary use of more traditional analytical tools such as UV/Vis, IR, NMR spectroscopy and mass spectrometry.

Practical problems such as very fast reactions, sample decomposition and reaction acceleration due to local heating effect [29], persists. A number of technological solutions for these problems have been developed. These include stopped-flow freeze-quenching to prolong the life-time of intermediates [30], and multiple-windows flow-reactors which enable steady-state observation of a reaction at different reaction time by controlling the flow-rate [8]. Many XAS studies on more challenging homogeneous catalytic reactions can therefore be expected in the near future.

## Abbreviations

Acac: Acetylacetate
bipy: 2,2’-Bipyridine
COD: 1,5-Cyclooctadiene
DMF: N,N-dimethylformamide
FTIR: Fourier-transformed infrared spectroscopy
BINOL: 1,1’-Bi-2-naphthol
EXAFS: Extended x-ray absorption fine structure spectroscopy
HERFD-XAS: High-energy resolution fluorescence detected XAS
HRMS: High resolution mass spectrometry
LCA: Linear combination analysis
NMR: Nuclear magnetic resonance spectroscopy
TMEDA: Tetramethylethylenediamine
UV/Vis: Ultraviolet/Visible spectroscopy
XANES: X-ray absorption near edge spectroscopy
XAS: X-ray absorption spectroscopy
